# Triglyceride-glucose Index and Mortality in a Large Regional-based Italian Database (URRAH Project)

**DOI:** 10.1210/clinem/dgae170

**Published:** 2024-03-14

**Authors:** Lanfranco D’Elia, Maria Masulli, Agostino Virdis, Edoardo Casiglia, Valerie Tikhonoff, Fabio Angeli, Carlo Maria Barbagallo, Michele Bombelli, Federica Cappelli, Rosario Cianci, Michele Ciccarelli, Arrigo F G Cicero, Massimo Cirillo, Pietro Cirillo, Raffaella Dell’Oro, Giovambattista Desideri, Claudio Ferri, Loreto Gesualdo, Cristina Giannattasio, Guido Grassi, Guido Iaccarino, Luciano Lippa, Francesca Mallamaci, Alessandro Maloberti, Stefano Masi, Alberto Mazza, Alessandro Mengozzi, Maria Lorenza Muiesan, Pietro Nazzaro, Paolo Palatini, Gianfranco Parati, Roberto Pontremoli, Fosca Quarti-Trevano, Marcello Rattazzi, Gianpaolo Reboldi, Giulia Rivasi, Elisa Russo, Massimo Salvetti, Giuliano Tocci, Andrea Ungar, Paolo Verdecchia, Francesca Viazzi, Massimo Volpe, Claudio Borghi, Ferruccio Galletti

**Affiliations:** Department of Clinical Medicine and Surgery, “Federico II” University of Naples, 80131 Naples, Italy; Department of Clinical Medicine and Surgery, “Federico II” University of Naples, 80131 Naples, Italy; Department of Clinical and Experimental Medicine, University of Pisa, 56126 Pisa, Italy; Studium Patavinum, Department of Medicine, University of Padua, 35100 Padua, Italy; Department of Medicine, University of Padua, 35100 Padua, Italy; Department of Medicine and Technological Innovation (DiMIT), University of Insubria, 21100 Varese, Italy; Department of Medicine and Cardiopulmonary Rehabilitation, Maugeri Care and Research Institutes, IRCCS, Tradate, 21049 VA, Italy; Biomedical Department of Internal Medicine and Specialistics, University of Palermo, 90100 Palermo, Italy; Internal Medicine, Pio XI Hospital of Desio, ASST Brianza, Department of Medicine and Surgery, University of Milano-Bicocca, Desio, 20832 MB, Italy; Department of Clinical and Experimental Medicine, University of Pisa, 56126 Pisa, Italy; Department of Translational and Precision Medicine, Sapienza University of Rome, 00185 Rome, Italy; Department of Medicine and Surgery, University of Salerno, Baronissi, 84081 SA, Italy; Hypertension and Cardiovascular Disease Research Center, Medical and Surgical Sciences Department, Alma Mater Studiorum University of Bologna, 40126 Bologna, Italy; Cardiovascular medicine unit, Heart-Chest-Vascular Department, IRCCS Azienda Ospedaliero-Universitaria di Bologna, 40126 Bologna, Italy; Department of Public Health, “Federico II” University of Naples, 80131 Naples, Italy; Nephrology, Dialysis and Transplantation Unit, Department of Emergency and Organ Transplantation, “Aldo Moro” University of Bari, 70122 Bari, Italy; Clinica Medica, Department of Medicine and Surgery, University of Milano-Bicocca, 20900 Monza, Italy; Department of Clinical, Internal Medicine, Anesthesiologic and Cardiovascular Sciences, Sapienza University of Rome, 00161 Rome, Italy; Department of Life, Health and Environmental Sciences, University of L’Aquila, 67100 L’Aquila, Italy; Nephrology, Dialysis and Transplantation Unit, Department of Emergency and Organ Transplantation, “Aldo Moro” University of Bari, 70122 Bari, Italy; Cardiology IV, “A.De Gasperi’s” Department, Niguarda Ca’ Granda Hospital, 20162 Milan, Italy; Department of Medicine and Surgery, School of Medicine and Surgery, Milano-Bicocca University, 20126 Milan, Italy; Clinica Medica, Department of Medicine and Surgery, University of Milano-Bicocca, 20900 Monza, Italy; Department of Clinical Medicine and Surgery, “Federico II” University of Naples, 80131 Naples, Italy; Italian Society of General Medicine (SIMG), 67051 Avezzano, Italy; Department of Nephrology, Dialysis and Transplantation GOM “Bianchi-Melacrino-Morelli” and CNR-IFC, Institute of Clinical Physiology, Research Unit of Clinical Epidemiology and Physiopathology of Renal Diseases and Hypertension (European Society of Hypertension, ESH, Excellence Centre) of Reggio Calabria, 89124 Reggio Calabria, Italy; Cardiology IV, “A.De Gasperi’s” Department, Niguarda Ca’ Granda Hospital, 20162 Milan, Italy; Department of Medicine and Surgery, School of Medicine and Surgery, Milano-Bicocca University, 20126 Milan, Italy; Department of Clinical and Experimental Medicine, University of Pisa, 56126 Pisa, Italy; Department of Internal Medicine, Santa Maria Della Misericordia General Hospital, AULSS 5 Polesana, 45100 Rovigo, Italy; Department of Clinical and Experimental Medicine, University of Pisa, 56126 Pisa, Italy; Center for Translational and Experimental Cardiology, Department of Cardiology, University Hospital Zurich, University of Zurich, 8952 Schlieren, Switzerland; Scuola Superiore Sant’Anna, 56127 Pisa, Italy; Department of Clinical and Experimental Sciences, University of Brescia, 25121 Brescia, Italy; Department of Precision and Regenerative Medicine and Jonic Area, Neurosciences and Sense Organs, University of Bari Medical School, 70122 Bari, Italy; Studium Patavinum, Department of Medicine, University of Padua, 35100 Padua, Italy; Department of Cardiology, S.Luca Hospital, IRCCS, Istituto Auxologico Italiano, 20149 Milan, Italy; Department of Medicine and Surgery, University of Milano-Bicocca, 20126 Milan, Italy; Dipartimento di Medicina Interna e Specialità Mediche, Università degli Studi di Genova, 16132 Genova, Italy; IRCCS Ospedale Policlinico San Martino, 16132 Genova, Italy; Clinica Medica, Department of Medicine and Surgery, University of Milano-Bicocca, 20900 Monza, Italy; Department of Medicine-DIMED, University of Padova, Medicina Interna 1°, Ca’ Foncello University Hospital, 31100 Treviso, Italy; Department of Medicine and Surgery, University of Perugia, 06100 Perugia, Italy; Department of Geriatric and Intensive Care Medicine, Careggi Hospital, University of Florence, 50121 Florence, Italy; Dipartimento di Medicina Interna e Specialità Mediche, Università degli Studi di Genova, 16132 Genova, Italy; IRCCS Ospedale Policlinico San Martino, 16132 Genova, Italy; Department of Clinical and Experimental Sciences, University of Brescia, 25121 Brescia, Italy; Department of Clinical and Molecular Medicine, University of Rome Sapienza, Sant’Andrea Hospital, 00189 Rome, Italy; Department of Geriatric and Intensive Care Medicine, Careggi Hospital, University of Florence, 50121 Florence, Italy; Department of Cardiology, Hospital S. Maria della Misericordia, 06100 Perugia, Italy; Dipartimento di Medicina Interna e Specialità Mediche, Università degli Studi di Genova, 16132 Genova, Italy; IRCCS Ospedale Policlinico San Martino, 16132 Genova, Italy; Dipartimento di Medicina Clinica e Molecolare, Università di Roma Sapienza, 00189 Roma, Italy; IRCCS San Raffaele Roma, 00163 Roma, Italy; Hypertension and Cardiovascular Disease Research Center, Medical and Surgical Sciences Department, Alma Mater Studiorum University of Bologna, 40126 Bologna, Italy; Cardiovascular medicine unit, Heart-Chest-Vascular Department, IRCCS Azienda Ospedaliero-Universitaria di Bologna, 40126 Bologna, Italy; Department of Clinical Medicine and Surgery, “Federico II” University of Naples, 80131 Naples, Italy

**Keywords:** triglyceride-glucose index, cardiovascular mortality, all-cause mortality, insulin resistance, uric acid

## Abstract

**Purpose:**

Recently, a novel index [the triglyceride-glucose (TyG) index]) was considered a surrogate marker of insulin resistance (IR); in addition, it was estimated to be a better expression of IR than widely used tools. Few and heterogeneous data are available on the relationship between this index and mortality risk in non-Asian populations. Therefore, we estimated the predictive role of baseline TyG on the incidence of all-cause and cardiovascular (CV) mortality in a large sample of the general population. Moreover, in consideration of the well-recognized role of serum uric acid (SUA) on CV risk and the close correlation between SUA and IR, we also evaluated the combined effect of TyG and SUA on mortality risk.

**Methods:**

The analysis included 16 649 participants from the URRAH cohort. The risk of all-cause and CV mortality was evaluated by the Kaplan–Meier estimator and Cox multivariate analysis.

**Results:**

During a median follow-up of 144 months, 2569 deaths occurred. We stratified the sample by the optimal cut-off point for all-cause (4.62) and CV mortality (4.53). In the multivariate Cox regression analyses, participants with TyG above cut-off had a significantly higher risk of all-cause and CV mortality than those with TyG below the cut-off. Moreover, the simultaneous presence of high levels of TyG and SUA was associated with a higher mortality risk than none or only 1 of the 2 factors.

**Conclusion:**

The results of this study indicate that these TyG (a low-cost and simple, noninvasive marker) thresholds are predictive of an increased risk of mortality in a large and homogeneous general population. In addition, these results show a synergic effect of TyG and SUA on the risk of mortality.

Cardiovascular (CV) diseases are the most common cause of mortality and morbidity in the general population (1). Insulin resistance (IR) is a condition associated with an increased risk of CV disease and type 2 diabetes mellitus (2, 3). Early identification of subjects affected by IR is important to classify people at high CV risk, for which therapeutic strategies should be intensified. The hyperinsulinemic-euglycemic clamp is the gold standard technique for assessing IR (4). However, due to the complexity of the method, it is only used in small-case research and not for population studies. Simpler methods have been proposed to be used in clinical practice and experimental studies conducted on larger samples. The Homeostatic Model Assessment of Insulin Resistance index is a simple, validated method, strongly associated with the hyperinsulinemic-euglycemic clamp: it is the marker of IR currently most widely used in clinical practice and epidemiological studies (4). Several other indices have been proposed to assess IR, among which are the Quantitative Insulin Sensitivity Check Index, Matsuda index, and McAuley index (5). All these tools require insulin level measurement, and this represents a limitation because insulin determination is not routinely performed in clinical practice and is relatively costly.

More recently, a new index has been developed to address these limitations: the triglyceride-glucose (TyG) index is simple, convenient, and low-cost because it does not require insulin measurement (6, 7). It has high sensitivity and specificity compared to the hyperinsulinemic-euglycemic clamp (7) and performs better than the Homeostatic Model Assessment of Insulin Resistance (8). The TyG is predictive of diabetes onset and better than fasting plasma glucose or triglycerides alone (9). Moreover, the results of a meta-analysis showed that TyG was directly associated with the risk of coronary artery disease and CV disease, while inconsistent results on all-cause and CV mortality in the general population were found (10). Of note, few and heterogeneous data are available on the relationship between this novel index and mortality risk in non-Asian populations (10-13).

Therefore, in consideration of these premises and that no TyG threshold has been proposed to identify people at high CV risk, this study aimed to evaluate the association of the TyG index with all-cause and CV mortality in the URic acid Right for heArt Health (URRAH) population to identify the better TyG threshold predictive for all-cause and CV mortality.

In addition, we also evaluated the combined effect of high TyG and high serum uric acid (HSUA) on the risk of mortality, in consideration of the well-recognized predictive role of serum uric acid (SUA) on CV risk (14), the strong independent predictive role of SUA on all-cause and CV mortality in the URRAH study (specifically designed to study the relationship between SUA and CV risk) both alone and in interaction with IR (15-17), and the close correlation between SUA and IR (18).

## Materials and Methods

### Study Population

The URRAH database is a multicenter retrospective, observational cohort study, which involves data from several cohorts recruited within Italian hypertension centers and distributed in almost all the Italian regions (age: 18-95 years). Full details of the URRAH project have been published previously (15). The URRAH study was performed according to the Declaration of Helsinki for Human Research (41st World Medical Assembly, 1990). Approval was sought from the ethics committee of the coordinating center at the Division of Internal Medicine of the University of Bologna (no. 77/2018/Oss/AOUBo). Informed consent was obtained from all individuals upon recruitment.

This study was planned, conducted, and reported according to the STROBE statement (https://www.equator-network.org/reporting-guidelines/strobe/) [Supplementary Table S1 (19)].

For the present study, 16 649 participants were considered, after the exclusion of participants without a complete database (n = 9192) and those who had only 1 year of observation to reduce a potential bias on mortality rate due to pre-existing diseases (n = 1237).

#### Examination procedures

The URRAH study procedures have been extensively described (15). Briefly, a nationwide Italian database was constructed by collecting individual data on patients with anthropometric and biochemical measurements, blood pressure (BP) and heart rate assessment, and clinical history information. Hypertension was defined as office systolic BP ≥ 140 and/or diastolic BP ≥90 mmHg or current antihypertensive drug treatment. Diabetes mellitus was defined according to standardized criteria (17). The estimated glomerular filtration rate (eGFR) was calculated by standard formula (20). The TyG index was calculated using the validated formula: Ln [TG (mg/dL)×fasting glucose (mg/dL)]/2 (21). HSUA was defined based on the previously described URRAH cut-off levels (ie, all-cause mortality: SUA >4.7 mg/dL; CV mortality: SUA >5.6 mg/dL) (15).

### Outcomes Assessment

All-cause and CV mortality were evaluated at the end of the follow-up. Information on patients who had died was obtained from hospital records or death certificates. Mortality from CV disease was coded according to the International Classification of Diseases, Tenth Revision (15).

#### Statistical analysis

All statistical analyses were performed using the SPSS software (version 23—SPSS Inc., Chicago, IL) and the statistical package R (version 4.3.1).

Because eGFR, SUA, total cholesterol, high-density lipoprotein (HDL)-cholesterol, low-density lipoprotein (LDL)-cholesterol, and heart rate were nonnormal distributed, log-transformed values were used in the analyses. Bivariate relationships between the variables under investigation were evaluated by Pearson's correlation analysis. The Chi-squared test was used to evaluate differences between categorical variables. To analyze the type of association between TyG (as a continuous variable) and mortality, restricted cubic splines regression models with 4 knots (5th reference, 35th, 65th, and 95th percentiles) were utilized.

Furthermore, the receiver-operating characteristic (ROC) analysis was carried out and the area under the curve (AUC), with its 95% confidence interval (CI), was calculated to assess the ability of TyG to identify participants who died for all-cause or CV event at follow-up. Next, the optimal cut-off point (Youden's index) of the association of continuous TyG with all-cause or CV mortality was identified by ROC analysis (all-cause mortality: 4.62; CV mortality: 4.53). According to these cut-off points, the sample was stratified into 2 groups and separately analyzed. The ANOVA for continuous data and the Chi-squared test to evaluate differences between categorical variables were used to evaluate statistical differences between groups’ characteristics. To analyze the predictive role of baseline TyG (as a dichotomous variable) on the risk of all-cause or CV mortality Kaplan–Meier survival curves, log-rank tests, and Cox proportional-hazards models were used. The impact of traditional risk factors was explored by multivariate models adjusted for baseline age, sex, hypertension status diabetes, eGFR, body mass index (BMI), cigarette smoking, heart rate, total cholesterol, HDL-cholesterol, statin use, therapy for high triglycerides, LDL-cholesterol and alcohol use. The proportional hazard assumption was assessed by visual inspection of Kaplan–Meier curves.

Finally, we simultaneously stratified the sample into 4 groups by TyG (cut-off all-cause mortality: 4.62; cut-off CV mortality: 4.53) and SUA [cut-off all-cause mortality: 4.7 mg/dL; cut-off CV mortality: 5.6 mg/dL (15)] to evaluate the interaction of the 2 factors in assessing mortality risk.

The results are reported as mean (or geometric mean) with SD, percentages, or hazard ratio (HR) and 95% CI (bootstrap CI, 1000 iterations), unless otherwise indicated. Two-sided *P*-values below .05 were considered statistically significant.

## Results

At baseline, the mean age of the whole sample (n = 16 649) was 56.0 years; 50% were men, 43% of the participants were overweight, 17% were obese, 70% were hypertensive (37% on regular antihypertensive treatment), 11% were diabetic, 25% were smokers, and 63% consumed alcohol. Four percent of the participants were on treatment with statins. The baseline TyG average was 4.62 (median: 4.61, SD: 0.29).

The analysis of the correlation between TyG and the most relevant characteristics of participants at baseline showed a significant and positive association with age (r = 0.21), BMI (r = 0.30), waist circumference (r = 0.42), systolic BP (r = 0.23), diastolic BP (r = 0.20), total cholesterol (r = 0.33), LDL-cholesterol (r = 0.14), SUA (r = 0.31), and heart rate (r = 0.09); while a significant and inverse association with eGFR (r = −0.22) and HDL (r = −0.33) was detected.

During a median follow-up of 144 months (25th-75th: 90-192 months), 2569 (15.4%) all-cause deaths occurred, 1124 of which were due to CV causes.

**Figure 1.**
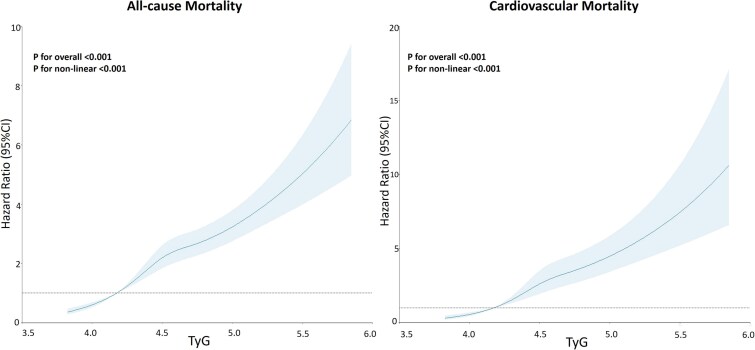
Association between the triglyceride-glucose index and risk of all-cause mortality and cardiovascular mortality using a restricted cubic spline regression model. Solid lines indicate hazard ratios, and shadow shapes indicate 95% confidence intervals.

Restricted cubic splines regression model detected a positive nonlinear relationship between TyG and all-cause and CV mortality (test for overall: *P* < .001, test for nonlinearity: *P* < .001) (Fig. 1).

Next, the AUCs for the relationship between TyG and all-cause and CV mortality were created. The AUC evaluation showed a significant ability to detect both all-cause (AUC and 95% CI: 0.60, 0.58-0.61: *P* < .001) and CV mortality (AUC and 95% CI: 0.61, 0.60-0.63: *P* < .001).

Given the stratification based on the optimal cut-off point by the ROC curve, we evaluated the predictive role of TyG for all-cause (>4.62) and CV mortality (>4.53) separately.

The TyG group with a value >4.62 had higher baseline age, BMI, waist circumference, BP, serum glucose, total cholesterol, triglycerides and LDL-cholesterol and lower renal function and HDL than those with ≤ 4.62 (Table 1). Similarly, the TyG group with a value >4.53 had higher baseline age, anthropometric indices, and BP; worse metabolic profile; and lower renal function than those with ≤ 4.53 (Table 1).

**Table 1.** Baseline characteristics of all the study participants stratified according to TyG index threshold predictive for all-cause mortality and cardiovascular mortality (n = 16 649)

Variables	TyG			
	All-cause mortality		Cardiovascular mortality	
	4.62≤	>4.62	4.53≤	>4.53
No. of participants (%)	51.5	48.5	38.7	61.3
Age (yrs)	53.5 (15.9)	58.7 (14.2)*^a^*	52.3 (16.2)	58.4 (14.3)*^b^*
Sex (M/F—%)	47.2/52.8	52.7/47.3*^a^*	45.9/54.1	52.3/47.7*^b^*
Cigarette smoking (%)	22.9	27.4*^a^*	22.4	26.8*^b^*
Alcohol consumption (%)*^c^*	57.9	68.0*^a^*	55.4	67.4
BMI (kg/m^2^)	25.3 (4.0)	27.5 (4.1)*^a^*	25.0 (4.0)	27.2 (4.1)*^b^*
Normal weight (%)	51.2	27.8	54.9	30.3
Overweight (%)	37.1	49.0	34.7	48.0
Obesity (%)	11.7	23.3*^a^*	10.4	21.7*^b^*
Waist circumference (cm)*^d,e^*	86.4 (12.7)	95.6 (11.5)*^a^*	85.2 (12.6)	94.6 (11.8)*^b^*
Systolic BP (mmHg)	141.2 (23.3)	150.3 (23.6)*^a^*	139.4 (23.1)	149.6 (23.5)*^b^*
Diastolic BP (mmHg)	85.0 (12.7)	89.2 (12.5)*^a^*	84.1 (12.7)	88.9 (12.5)*^b^*
Hypertension (%)	61.8	78.4*^a^*	58.2	77.2*^b^*
Heart rate (b/min)*^d,f^*	70.8 (1.2)	72.4 (1.2)*^a^*	70.8 (1.2)	72.4 (1.2)*^b^*
eGFR (mL/min/1.73 m^2^)*^d^*	85.1 (1.3)	75.8 (1.3)*^a^*	87.1 (1.3)	77.6 (1.3)*^b^*
Serum uric acid (mg/dL)*^d^*	4.5 (1.3)	5.3 (1.3)*^a^*	4.5 (1.3)	5.1 (1.3)*^b^*
Glucose (mg/dL) *^d,f^*	89.1 (1.1)	104.7 (1.2)*^a^*	89.1 (1.1)	102.3 (1.2)*^b^*
Diabetes (%)	4.2	17.9*^a^*	3.6	15.4*^b^*
Total cholesterol (mg/dL)*^d,f^*	195.0 (1.2)	218.8 (1.2)*^a^*	195.0 (1.2)	218.8 (1.2)*^b^*
Triglycerides (mg/dL)*^d,g^*	74.1 (1.3)	162.2 (1.4)*^a^*	67.6 (1.3)	144.5 (1.5)*^b^*
Therapy for hypertriglyceridemia (%)	0.1	1.3*^a^*	0.1	1.0*^b^*
HDL cholesterol (mg/dL)*^d,h^*	54.9 (1.3)	46.8 (1.3)*^a^*	55.0 (1.3)	47.9 (1.3)*^b^*
LDL cholesterol (mg/dL)*^d,h^*	123.0 (1.3)	134.9 (1.3)	120.2 (1.3)	134.9 (1.3)
Statin use (%)	3.2	4.6*^a^*	2.8	4.6*^b^*

Data are expressed as means (SD). Overweight was defined as a BMI between 25 and 29.9 kg/m^2^ and obesity as BMI ≥ 30 kg/m^2^. Hypertension was defined as office systolic BP ≥ 140 and/or diastolic BP ≥90 mmHg or current antihypertensive drug treatment.

Abbreviations: BMI, body mass index; BP, blood pressure; eGFR, estimated glomerular filtration rate; HDL, high-density lipoprotein; LDL, low-density lipoprotein; TyG, triglyceride-glucose; WC, waist circumference.

^*a*^Above 4.62 vs below 4.62: *P* < .05.

^*b*^Above 4.53 vs below 4.53: *P* < .05

^*c*^Sample reduced by 40%.

^*d*^Data are expressed as geometric mean (SD).

^*e*^Sample reduced by 50%.

^*f*^Sample reduced by 10%.

^*g*^Sample reduced by 6%.

^*h*^Sample reduced by 20%.

Participants with TyG more than 4.62 had a higher incidence of all-cause mortality than participants with TyG below 4.62 (18.9% vs 12.1%, *P* < .0001). Likewise, participants with TyG more than 4.53 had a higher incidence of CV mortality than participants with TyG below 4.53 (8.4% vs 4.2%, *P* < .0001).

**Figure 2.**
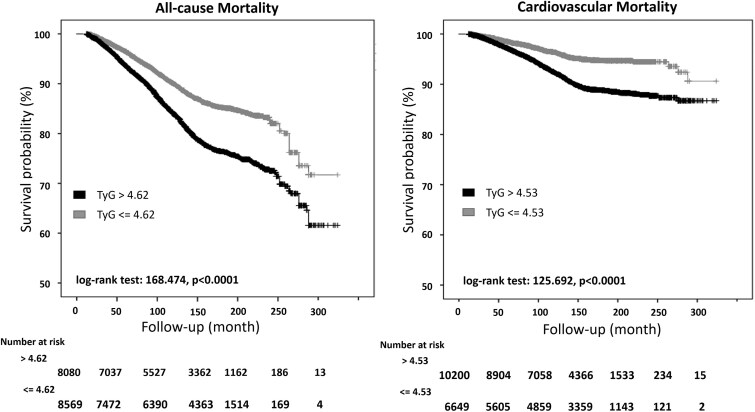
Kaplan–Meier curves for all-cause mortality for people with triglyceride-glucose index lower or above 4.62 and cardiovascular mortality for people with triglyceride-glucose index lower and above 4.53.

The Kaplan–Meier curves for all-cause and CV mortality are shown in Fig. 2. In particular, participants with values above 4.62 had a significantly higher probability of all-cause mortality than those with TyG below 4.62 (log-rank test: 168.474, *P* < .0001), and those with values above 4.53 had a significantly higher probability of CV mortality than those with TyG below 4.53 (log-rank test: 125.692, *P* < .0001). The inspection of Kaplan–Meier curves did not detect a proportional hazard assumption violation. Cox-regression analysis confirmed the predictive role of all-cause mortality cut-off, which showed a greater risk of all-cause mortality in participants with TyG above with respect to below 4.62 (HR: 1.67, 95% CI: 1.55-1.81). This predictive role was also detected after adjustment for some potential confounders (Table 2). A similar trend was also found for CV mortality; in particular, participants with TyG above vs below 4.53 had a significantly higher risk both in unadjusted (HR: 2.15, 95% CI: 1.87-2.46) and adjusted models (Table 2).

**Table 2.** Cox-regression analysis of risk of all-cause and cardiovascular mortality according to the 2 different thresholds of the TyG index

	All-cause mortality	Cardiovascular mortality
	TyG > 4.62 vs TyG ≤ 4.62	TyG > 4.53 vs TyG ≤ 4.53
	HR (95% CI)	HR (95% CI*^a^*)
Unadjusted	1.67 (1.55-1.81)	2.15 (1.87-2.46)
Multivariable model 1*^b^*	1.23 (1.13-1.34)	1.43 (1.24-1.65)
Multivariable model 2*^c^*	1.12 (1.03-1.22)	1.29 (1.11-1.49)
Multivariable model 3*^d^*	1.10 (1.01-1.20)	1.24 (1.07-1.44)
Multivariable model 4*^e^*	1.22 (1.11-1.34)	1.37 (1.17-1.60)
Multivariable model 5*^f^*	1.20 (1.09-1.34)	1.36 (1.15-1.60)
Multivariable model 6*^g^*	1.12 (1.01-1.25)	1.26 (1.06-1.49)

Abbreviations: BMI, body mass index; CI, confidence interval; eGFR, estimated glomerular filtration rate; HR, hazard ratio; SUA, serum uric acid; TyG, triglyceride-glucose.

^*a*^Bootstrap confidence intervals (1000 iterations).

^*b*^Model 1 adjusted for baseline age, sex, BMI, hypertension, statin use, (log)SUA.

^*c*^Model 2 adjusted for model 1 plus baseline diabetes.

^*d*^Model 3 adjusted for model 2 plus baseline (log)eGFR, cigarette smoking.

^*e*^Model 4 adjusted for model 3 plus baseline (log)total cholesterol, therapy for hypertriglyceridemia (sample reduced by 10%).

^*f*^Model 5 adjusted for model 4 plus baseline (log)high-density lipoprotein-cholesterol and (log) heart rate (sample reduced by 20%).

^*g*^Model 6 adjusted for model 2 plus baseline therapy for hypertriglyceridemia, (log) heart rate, (log)low-density lipoprotein-cholesterol and alcohol consumption (sample reduced by 40%).

Furthermore, a separate analysis on the comparison between the predictive value of TyG and that of classical risk factors indicated a higher AUC of TyG than those of SUA, BMI, total cholesterol, and eGFR but lower than those of age and systolic BP [Supplementary Table S2 (22)].

**Figure 3.**
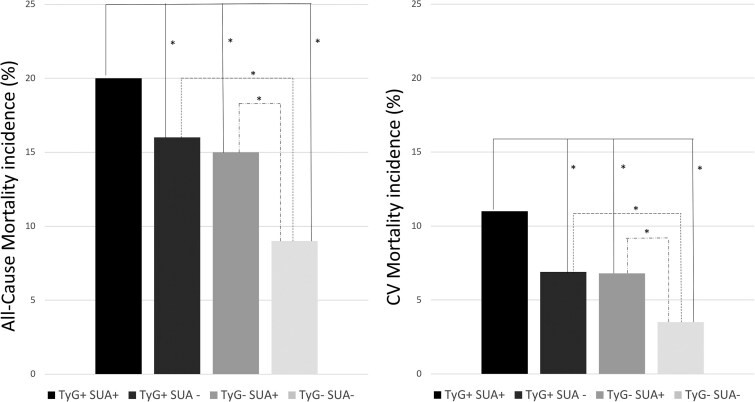
The incidence rate of all-cause and cardiovascular mortality in subjects stratified by TyG index and HSUA. All-cause mortality, TyG+: >4.62, TyG−: ≤ 4.62, HSUA+: >4.7 mg/dL, HSUA−: ≤4.7 mg/dL. Cardiovascular mortality, TyG+: >4.53, TyG−: ≤4.53, HSUA+: >5.6 mg/dL, HSUA−: ≤5.6 mg/dL. *P < .05. Abbreviations: HSUA, high serum uric acid; TyG, triglyceride-glucose.

Finally, we analyzed the predictive role of TyG with SUA interaction on all-cause and CV mortality. The percentage of participants who died from all-cause or CV events was significantly higher among those with greater TyG and HSUA than in other groups (*P* < .001) (Fig. 3). This trend was also confirmed by Cox-regression analysis, which also highlighted a slight nonsignificant higher mortality risk of the high TyG group than the high SUA group (Table 3).

**Table 3.** Risk of mortality by the TyG index and serum uric acid

	All-cause mortality	Cardiovascular mortality
	HR (95% CI*^a^*)	HR (95% CI*^a^*)
TyG+/HSUA+	2.36 (2.11-2.64)	3.35 (2.81-3.99)
TyG+/HSUA−	1.87 (1.63-2.15)	2.11 (1.77-2.52)
TyG−/HSUA+	1.68 (1.48-1.90)	2.07 (1.61-2.66)
TyG−/HSUA−	reference	reference
Other comparisons		
TyG+/HSUA+ vs TyG−/HSUA+	1.41 (1.28-1.55)	1.62 (1.29-2.02)
TyG+/HSUA+ vs TyG+/HSUA−	1.26 (1.12-1.42)	1.59 (1.39-1.82)
TyG−/HSUA+ vs TyG+/HSUA−	0.90 (0.79-1.02)	0.98 (0.78-1.23)

All-cause mortality: TyG+ >4.62, TyG**−** ≤ 4.62; cardiovascular mortality: TyG+ > 4.53, TyG**−** ≤ 4.53); HSUA: all-cause mortality: HSUA+ >4.7 mg/dL, HSUA**−** ≤4.7 mg/dL; cardiovascular mortality: HSUA+ >5.6 mg/dL, HSUA**−** ≤5.6 mg/dL.

Abbreviations: CI, confidence interval; HR, hazard ratio; HSUA, high serum uric acid; TyG, triglyceride-glucose.

^*a*^Bootstrap confidence intervals (1000 iterations).

## Discussion

The results of this study show that TyG is a significant predictor of all-cause and CV mortality in the general population. In particular, our study indicates that TyG is positively and nonlinearly associated with mortality risk: values more than 4.62 were associated with a 1.69-fold increased risk of all-cause mortality than lower, and TyG more than 4.53 was associated with a 2-fold increased risk of CV mortality than lower values, independently of potential confounders (such as age, hypertension, diabetes, smoking habits, BMI, renal function, and lipid profile).

Although TyG has recently been assessed, several observational studies explored the predictive role of mortality and CV risk. A recent meta-analysis detected that a higher TyG was associated with coronary artery disease, myocardial infarction, and CV disease compared to a lower TyG (9). By contrast, this meta-analysis found inconsistent results on all-cause and CV mortality in the general population. However, the analysis included few studies (4 for all-cause and 3 for CV mortality), and only 1 of them reported results of a non-Asian cohort (10). This latter study included a large American sample of men and showed an indirect significant association between TyG and all-cause mortality and an indirect not significant association with CV mortality (12). On the other hand, a more recent study on 2 large Swedish general populations found a direct and significant association between TyG and all-cause or CV mortality (13). In line with these data, the recent results of the PURE study (a large prospective study including 22 countries across 5 continents) showed that TyG was significantly associated with CV mortality, incident CV diseases, and type 2 diabetes mellitus (11). Notably, the study of Vega et al (12) considered a cohort of only men, with a TyG index not comparable with ours [ie, different units of measurement (mg^2^)], and a potential bias in the analysis due to the collinearity between TyG and non-HDL cholesterol included in the same model, while the studies of Muhammad et al (13) and Lopez-Jaramillo et al (11) did not detect any cut-off. Therefore, the results of our study have advantages with respect to those who did not detect any cut-offs, because our data can be easily included in the stratification of the CV risk and applicable to the clinical practice.

Noteworthy, in the literature the mean values of the TyG index are reported using 2 ranges: approximately from 4 to 5 and from 8 to 9. This difference derives from a misunderstanding of the original formula, which divides by 2 the natural results of the logarithm, whereas the alternative formula reports the natural logarithm of the product (triglyceride × glucose) divided by 2 (21). Nonetheless, the 2 ranges do not generate a difference in the relationship between the index and outcomes. However, having to validate a cut-off, we adopted the original range in this study (6).

Our analysis indicated a worse cardiometabolic profile in those who had TyG values above the thresholds. This result may in part explain the higher mortality risk in participants with a greater TyG than lower values. Moreover, since TyG is an indicator of IR, it is expected to have an unfavorable association with several CV risk factors (eg, diabetes, excess body weight, BP, arterial stiffness) (23). Indeed, in support of this notion, we also found a direct correlation between TyG and anthropometric indices, BP, SUA, and lipid profile and an inverse correlation with renal function and HDL. Different mechanisms support these associations; for example, IR provokes low-grade inflammation, endothelial dysfunction, and arterial stiffness (23) and, also by the interaction with leptin (24), may activate the sympathetic nervous system and alter renal sodium handling (23).

Furthermore, our data pointed out on potential interaction between TyG and SUA, with IR as a common denominator. The role of SUA as a CV risk factor and a metabolic mediator is well recognized, indeed the European guidelines for the management of hypertension recommend SUA evaluation in the stratification of the CV risk of patients with arterial hypertension (14). In particular, in this context, the URRAH study showed a univocal predictive role of SUA to predict all-cause and CV mortality and CV events and diseases in different settings (15-17).

Several studies detected a positive association between TyG and SUA in different populations (25-27), in addition to much data on that between SUA and IR (28). For instance, insulin can regulate the reabsorption of tubular uric acid (29); SUA may affect insulin signaling and promote alteration of the glucose metabolism by increasing the production of reactive oxygen species (30); and, vice versa, IR may provoke increased SUA by promoting inflammation (30). The results of our analysis confirmed this relationship: the simultaneous presence of high levels of TyG and high levels of SUA was associated with a higher risk of mortality with respect to none or only 1 of the 2 factors. Moreover, a slight nonsignificant higher mortality risk was found for high levels of TyG compared with high SUA.

This study has several strengths: the large and homogeneous sample size; the long follow-up period that allows reaching a large number of events; the study population being highly representative of the general population; the soundness of the results; the identification of a threshold separately for all-cause and CV mortality; and a greater predictive role of TyG on mortality in respect to some classical risk factors (eg, BMI, LDL-cholesterol, and renal function) [Supplementary Table S2 (22)]. For these reasons, the results of this study make TyG thresholds applicable to the routine clinical practice of CV prevention and risk stratification.

Nevertheless, our study has some limitations: the study design is observational. Some variables, such as clinical history and drugs, were self-reported, and the potential influence of some unmeasured variables (eg, salt intake) cannot be excluded. Finally, the URRAH population comprises individuals of White ethnicity: whether these observations can also be extended to non-Caucasian ethnic groups remains to be determined.

## Conclusions

This study for the first time detected a TyG threshold for all-cause and CV mortality in the Caucasian general population. The results of the present study indicate that these thresholds are predictive of an increased risk of all-cause and CV mortality in a large and homogeneous general population. In particular, the threshold of 4.62 can predict all-cause mortality and 4.53 CV mortality: these cut-offs are able to identify individuals with the clinical phenotype of the metabolic alterations and so at higher mortality risk. Furthermore, these results suggest that TyG may serve as a low-cost and simple, noninvasive marker for CV risk stratification in the general population, rather than the more complex, expensive, and laborious assessments of IR. Lastly, these results confirm the strong relationship between IR and SUA and indicate an unfavorable synergic effect of high levels of TyG and high levels of SUA on the risk of mortality. Nevertheless, further studies are needed to support our conclusions.

## Data Availability

Some or all datasets generated during and/or analyzed during the current study are not publicly available but are available from the corresponding author on reasonable request.
